# Extracellular peptidases of the cereal pathogen *Fusarium graminearum*

**DOI:** 10.3389/fpls.2015.00962

**Published:** 2015-11-06

**Authors:** Rohan G. T. Lowe, Owen McCorkelle, Mark Bleackley, Christine Collins, Pierre Faou, Suresh Mathivanan, Marilyn Anderson

**Affiliations:** Department of Biochemistry and Genetics, La Trobe Institute for Molecular Science, La Trobe UniversityMelbourne, VIC, Australia

**Keywords:** *Fusarium graminearum*, peptidase, protease, orbi-trap, proteomics, secretome, fungi, plant-pathogen

## Abstract

The plant pathogenic fungus *Fusarium graminearum* (Fgr) creates economic and health risks in cereals agriculture. Fgr causes head blight (or scab) of wheat and stalk rot of corn, reducing yield, degrading grain quality, and polluting downstream food products with mycotoxins. Fungal plant pathogens must secrete proteases to access nutrition and to breakdown the structural protein component of the plant cell wall. Research into the proteolytic activity of Fgr is hindered by the complex nature of the suite of proteases secreted. We used a systems biology approach comprising genome analysis, transcriptomics and label-free quantitative proteomics to characterize the peptidases deployed by Fgr during growth. A combined analysis of published microarray transcriptome datasets revealed seven transcriptional groupings of peptidases based on *in vitro* growth, *in planta* growth, and sporulation behaviors. A high resolution mass spectrometry-based proteomics analysis defined the extracellular proteases secreted by *F. graminearum*. A meta-classification based on sequence characters and transcriptional/translational activity *in planta* and *in vitro* provides a platform to develop control strategies that target Fgr peptidases.

## Introduction

The first stages of the plant fungal-pathogen interaction occur on epidermal cells, followed by intercellular spaces such as the apoplastic space (Jones and Dangl, 2006). Proteins secreted by pathogens may dictate the interaction on several levels: (1) Degrading enzymes breakdown host macromolecules to provide nutrition for the pathogen (Brunner et al., 2013). (2) Toxin proteins actively disrupt cellular function of the host and kill cells (Ciuffetti et al., 2010). (3) Immune modulator proteins may inadvertently alert the host to pathogen attack preventing colonization, or conversely they may camouflage non-protein elicitors such as chitin to allow continued growth (Dodds and Rathjen, 2010). Indeed, the size and complexity of a fungal secretome is shaped by their lifestyle and ecological niche (Lowe and Howlett, 2012).

Plant pathogens must extract nutrition from the host plant during colonization, and this aspect of the interaction will be the basis of our study. Two of the key nutrients for fungal growth are carbon and nitrogen. Carbon, most often in the form of carbohydrate, is required as a source of cellular energy as well as for growth and remodeling. Nitrogen is required for synthesis of proteins and nucleic acids. Host-derived protein provides a major source of both carbon and nitrogen. These host proteins must be digested into short peptide fragments before import, and this digestion is performed by a suite of proteases that are secreted into the environment. Therefore, a key aspect of the host-pathogen interaction is this interplay between peptidases secreted by the pathogen and the host substrates.

The host is not a passive partner. It actively responds to signs of infection, such as damage-associated molecular patterns (DAMPS). These DAMPS may be created by the action of secreted proteins, for example when peptidase activity releases hydrophobic peptides that are normally sequestered in the native protein (Seong and Matzinger, 2004). Responses include deployment of a range of defense molecules that have evolved to prevent the fungus from establishing an infection. Defense strategies in the plant have been studied from a number of different aspects and there is an abundance of literature on the subject (Jones and Dangl, 2006). Less is known about the proteins that the fungus produces to invade the plant tissue, evade the immune response and utilize plant material as a source of carbon, nitrogen and other essential nutrients.

Research on the proteins secreted by the fungus as virulence factors has focused on small proteins described as fungal effectors, for example, avirulence proteins reviewed in Stergiopoulos and de Wit (2009). The role of peptidases in plant-pathogen interactions has been dominated by classical nutrient acquisition, or catabolic activities, but there are instances of peptidases determining the outcome of a plant-pathogen interaction by other mechanisms. The bacterial effector HopN1, from *Pseudomonas syringae*, is a cysteine peptidase that once secreted into the plant cell will cleave PsbQ, an essential photosynthesis enzyme, and block programmed cell death (Rodríguez-Herva et al., 2012). In addition, the AvrPphB, ORF4 and NopT effectors also from *P. syringae* cleave themselves following delivery into plant cells to expose peptides containing fatty acid acylation motifs (Dowen et al., 2009). Acylation of these sites controls targeting to the plant plasma membrane and their avirulence activity. Among the oomycetes, the soybean pathogen, *Phytophthora sojae*, secretes a class of endoglucanase inhibitor proteins (GIPs) (Rose et al., 2002). These proteins share sequence similarity with serine peptidases, yet lack proteolytic activity due to mutation of the catalytic triad. A final example is the Avr-Pita avirulence protein from the rice blast fungus *Magnaporthe oryzae*, which has sequence similarity to metallopeptidases in the M35 clan. This peptidase-like protein is secreted and subsequently detected by the rice receptor protein Pi-ta (Jia et al., 2000). This interaction triggers a host defense response and leads to host resistance. Studies on the human pathogen *Candida albicans* have revealed that proteomic analysis leads to the identification of secreted proteins that were not identified by other methods (Gil-Bona et al., 2015). Similarly, studies in the model fungus *Saccharomyces cerevisiae* revealed that many of the proteins present in the secretome lacked the typical signal sequences required for annotation as secreted molecules (Giardina et al., 2014). From this evidence, it is clear that a multi-faceted approach is needed to describe the full secretome of a fungal pathogen and define the role it plays in plant-pathogen interactions.

*Fusarium graminearum* (aka *Gibberella zeae*) is a widespread pathogen of cereals such as wheat, barley, and corn, which infects the floral tissues and stems of plants and causes major economic losses to growers (Goswami and Kistler, 2004). As well as reducing grain yields, *F. graminearum* produces mycotoxins such as deoxynivalenol in the infected grain that pollute food and feed supplies (Sobrova et al., 2010). Mycotoxin levels in grain are strictly monitored and the presence of mycotoxins severely restricts market options for growers. The burden of this disease was made starkly apparent by an epidemic of Fusarium head blight in the northern great plains and central USA region over the 1998–2000 seasons, which caused an estimated 2.7 billion dollars of economic impact (Nganje et al., 2002).

For nutrition to be accessible, high molecular weight molecules must be degraded with a variety of extracellular hydrolases to produce low molecular weight products for import into the cell. A full understanding of the proteins secreted by *F. graminearum* will provide key targets for control of this fungus. Indeed, peptidase inhibitors have already been reported to be upregulated in two wheat cultivars that are resistant to Fusarium head blight (Gottwald et al., 2012). Specifically, a Bowman-Birk type peptidase inhibitor (gene ID Ta.21350.2.S1, MEROPS family I12) was reported that is predicted to inhibit serine peptidases of the MEROPS S1 family. In addition, a gene encoding a subtilisin-like serine protease inhibitor (Ta.22614.1.S1) was specifically upregulated in the resistant cultivars “Dream” and “Sumai-3” during head blight infection. Knowledge of the fungal peptidase targets for these inhibitors may improve the selection of resistant wheat cultivars.

Over 20 studies have identified proteins from *Fusarium* as well as proteins of Triticum species and barley produced during infection of floral tissues, as reviewed by Yang et al. (2013). Among these, the most exhaustive range of conditions for protein production was reported by Paper et al. (2007). They identified 289 secreted *F. graminearum* proteins using a linear ion-trap quadrupole mass-spectrometer analysis of *in vitro* secreted proteins as well as extracts from infected plant tissues (Paper et al., 2007). Many studies were limited by either low protein abundance *in planta*, low sensitivity of 2D-gel formats, or low sample replication.

We set out to use a systems biology approach to define the extracellular proteome of *F. graminearum* to facilitate discovery of critical targets for control of diseases such as Gibberella stalk rot of corn, or Fusarium head blight of wheat. Here, we combined public gene expression data for *F. graminearum* with our own high sensitivity LTQ Orbitrap MS/MS analysis to extend the known secretome of this agriculturally important pathogen. As nitrogen is one of the key nutrients the fungus must release from the host plant, three different nitrogen sources were compared to reveal an extended range of secreted peptidases tailored to each condition.

## Methods

### Growth and maintenance of fungal cultures

*F. graminearum* 73B1A (kindly provided by Dr Gusui Wu, Pioneer Dupont, USA), was routinely cultured on synthetic nutrient poor agar (SNA) at 25°C with a 14 h light:dark cycle.

### Culture media for proteomics

*F. graminearum* 73B1A was grown in four different culture media for proteomics studies. Half-strength Difco potato dextrose broth (1/2 PDB) was used as a complex medium, and three defined media were prepared with differing nitrogen sources. The defined media are derivatives of Czapek-dox medium (Czapek, 1902, 1903; Dox, 1910) with varied nitrogen sources. The basal composition was glucose (10 g/L), di-potassium phosphate (1 g/L), magnesium sulfate (0.5 g/L), potassium chloride (0.5 g/L), and ferrous sulfate (0.01 g/L) at pH 5.1. Nitrogen was added to ensure a molar carbon:nitrogen ratio of 20:1, nitrate medium (NO_3_) contained sodium nitrate (16.5 mM, 1.4 g/L), glutamine medium contained glutamine (9 mM, 1.31 g/L), minus N medium had no nitrogen. 1/2 PDB was sterilized by autoclaving, while defined media were filter sterilized and used within 2 days.

### Culture conditions for proteomics

Samples for secreted proteomics analysis were created as follows. *F. graminearum* 73B1A conidia were added to 1/2 PDB (500 mL at 1.5 × 10^4^ spores/mL) and grown at 20°C and 85 rpm for 1 day. This master culture was split into 12 aliquots of 40 mL. The hyphae were collected by centrifugation (3220 g, 15 min), and were washed three times with the growth medium. Washed hyphae were resuspended in 50 mL of medium and grown for 1 day at 20°C, 85 rpm. The culture was filtered with a 0.22 μM low-bind VWSP filter disc (Millipore) to separate hyphae from culture medium. The filtrate was concentrated down to 4 mL with a 3 kDa ultrafiltration column (Amicon ultra, Millipore) prior to protein precipitation. Three biological replicates were prepared in parallel. A single cellular proteome control sample was prepared in the same way as the secreted proteome samples, except that hyphae were retained from the 1 day 1/2 PDB culture and were washed 3 × with sterile water before they were snap frozen in liquid nitrogen and freeze-dried. Dried hyphae (20 mg) were added to 400 μL of urea extraction buffer (1% SDS, 8 M urea, 10%glycerol, 25 mM Tris HCl pH6.8, 1 mM EDTA, 0.7 M mercapto-ethanol) and glass beads (0.5 mm dia.) equal to 1/4 the hyphal volume before homogenization at 30 Hz for 30 s in a mixer mill (Qiagen). The sample was boiled for 2 min and then cooled on ice. The recovered supernatant formed the cellular proteome crude extract.

### Precipitation of secreted proteins

Trichloroacetic acid (1 mL of 6.1 N) was added to 4 mL of crude secretome filtrate, mixed, and incubated at 4°C overnight. It was then centrifuged at 13,000 g for 20 min at 4°C to pellet the proteins. The supernatant was removed and the pellet was washed with 800 μL of ice-cold acetone, by vortexing, centrifugation (13,000 g, 20 min) and removal of the acetone. The acetone wash was repeated twice. Each acetone-washed pellet was agitated in 100 μL resuspension buffer (8 M urea, 10 mM dithiothreitol) at 30°C until fully dissolved, before protein levels were compared by image analysis after SDS-PAGE and SYPRO ruby staining. The cellular proteome was processed with the same method as the secretome except acetone was used in the initial precipitation instead of trichloroacetic acid.

### In solution trypsin digest for proteomics

Protein was precipitated from extracts with 5 volumes of acetone, washed in acetone, and resuspended in digest buffer (8 M urea, 50 mM ammonium bicarbonate, 10 mM DTT) before incubation at 37°C for 30 min. Iodoacetamide was then added to 55 mM to alkylate thiol groups (45 min, dark, and 20°C). The alkylated preparation was diluted to 1 M urea with 25 mM ammonium biocarbonate (pH 8.5) before sequencing grade trypsin (Promega) was added to 5 μM final concentration. Digests were performed overnight (37°C) with shaking to produce tryptic peptides. Tryptic peptides were acidified with 1% formic acid (v/v).

### Solid phase extraction clean-up of tryptic peptides

Tryptic peptides in 1% (v/v) formic acid were centrifuged at 18,000 rcf for 2 min before application to a solid phase extraction column (1cc Oasis HLB, Waters) that had been conditioned with 800 μL of buffer A [80% (v/v) acetonitrile, 0.1% (w/v) trifluroacetic acid], followed by 1000 μL of buffer B (0.1%trifluroacetic acid). After application of the tryptic peptides, the column was washedin buffer B, before the peptides were eluted in 800 μL of buffer A, and concentrated to 100 μL final volume for mass spectrometry analysis.

### ESI–LC–MS/MS

Peptides (2 μL) were diluted to 30 μL in 0.1% trifluroacetic acid and 2% acetonitrile (buffer A) were loaded onto a trap column (C18 PepMap 100 μm i.d. × 2 cm trapping column, Thermo-Fisher Scientific) at 5 μL/min for 6 min and washed for 6 min before switching the precolumn in line with the analytical column (Easy-Spray 75 μm i.d. × 50 cm, Thermo-Fisher Scientific). The separation of peptides was performed at 250 nL/min using a linear acetonitrile gradient of buffer A and buffer B (0.1% formic acid, 80% acetonitrile), starting from 5% buffer B to 60% over 300 min. Data were collected on an Orbitrap Elite (Thermo-Fisher Scientific) in Data Dependent Acquisition mode using m/z 300–1500 as MS scan range. CID MS/MS spectra were collected for the 20 most intense ions. Dynamic exclusion parameters were set as follows; repeat count 1, duration 90 s, the exclusion list size was set at 500 with early expiration disabled.

Other instrument parameters for the Orbitrap were as follows: MS scan at 120,000 resolution, maximum injection time 150 ms, AGC target 1 × 10^6^, CID at 35% energy for a maximum injection time of 150 ms with AGT target of 5000. The Orbitrap Elite was operated in dual analyser mode with the Orbitrap analyser being used for MS and the linear trap being used for MS/MS.

### Proteomics database searches

All searches were made against the *F. graminearum* PH-1 (FG3) predicted proteome annotation from the Broad institute (“Fusarium Comparative Sequencing Project, Broad Institute of Harvard and MIT”)^1^. Protein sequences from the cRAP database of common lab contaminants (www.thegpm.org/crap) were added to the database. Decoy sequences were included for all searches.

Label-free quantitation (LFQ) of protein abundance was performed with MaxQuant software and the Andromeda search engine (Cox et al., 2011, 2014). Default settings were used, with “Match between runs” and “requantify” turned on. Both PSM and Protein false discovery rates were set to 0.01. Search engine variable modifications parameters were: oxidized methionine, N-terminal acetylation. The fixed modifications used were: carbamidomethylation of cysteine, precursor ion mass tolerance of 20 ppm (initial search), 10 ppm (second search) and fragment ion mass tolerance of 0.5 Da.

High sensitivity qualitative searches of the cellular control MS/MS spectra were performed using Search GUI and Peptide shaker (Vaudel et al., 2015) within the Galaxy environment (Boekel et al., 2015). Input mgf peak lists were processed by X!Tandem, MS-GF+, and OMSSA using the same parameters as described above for MaxQuant. Searches were performed and combined with SearchGUI before being passed to Peptide shaker to process the output and produce a single combined analysis. Peptide shaker was run with a false discovery rate of 1% at the protein, peptide and PSM level. MzidentML files were created and protein reports exported from Peptide Shaker with the final protein identifications. Proteomics spectra files and protein identifications were deposited at the EBI PRIDE archive (http://www.ebi.ac.uk/pride/archive/) under project accessions PXD002786 and PXD002840.

### Bioinformatics

Microarray transcriptome datasets were downloaded from the PLexDB database (www.plexdb.org), experiments FG01, FG02, FG05, FG06, FG07, FG10, FG11, FG12, FG13, FG14, FG15, FG16, FG18, FG19 were used in this analysis, see Table 1 for a summary of the microarray details. RMA gene expression values were log base10 transformed and imported to the R statistical software environment. Clustering and heat map plots were performed using the heat.map.2 R module. Euclidean-distance complete-linkage trees were produced for each axis of the heat map.

**Table 1:** *****F. graminearum*** microarray transcriptomics resources**.

**PlexDB ID**	**Experiment description**	**Citation**
FG01	Barley head blight infection	Güldener et al., 2006
FG02	Carbon and Nitrogen starvation *in vitro*	Güldener et al., 2006
FG05	Sexual development *in vitro*	Hallen et al., 2007
FG06	Cch1 deletion mutant	Hallen and Trail, 2007
FG07	Asexual spore germination *in vitro*	Seong et al., 2008
FG10	Trichodiene treatment *in vitro*	Seong et al., 2009
FG11	Growth of TRI6, TRI10 deletion mutants	Seong et al., 2009
FG12	Wheat crown rot infection	Stephens et al., 2008
FG13	Growth of a StuA mutant	Lysøe et al., 2010
FG14	Deoxynivalenol induction *in vitro*	Gardiner et al., 2009
FG15	Wheat head blight infection	Lysøe et al., 2011
FG16	Sexual development on wheat	Guenther et al., 2009
FG18	Fg1p deletion mutant deoxynivalenol biosynthesis	Jonkers et al., 2012
FG19	Wheat coleoptile infection	Zhang et al., 2012

Sequence-based prediction of secretion was performed using a three stage process similar to a previously published example (Brown et al., 2012). Firstly, SignalP4.1 (Petersen et al., 2011) was used to predict signal peptide presence, secondly TmHMM2.0 (Krogh et al., 2001) was used to identify trans-membrane regions, and thirdly WolfPsort (Horton et al., 2007) was used to predict likely cellular location. To be included in our “high confidence secretion” cohort a sequence had to include a signal peptide, have no trans-membrane regions outside of the signal peptide, and score 17 or more for the “extracellular” classification on WolfPsort.

Principal components analysis (PCA) was performed using R software and the prcomp function. Briefly, MaxQuant label-free quantitation abundances (LFQ) for each replicate of the four secretome treatments were extracted from the MaxQuant proteinGroups.txt output file and used as input data. The input data matrix was Log(2) transformed and quantile normalized in R before PCA was performed on only the 134 protein high-confidence secretome. Missing values were substituted with the minimum value for that sample prior to PCA. Optional settings were left as the default settings, including: rotated variables, zero centered, no scaling.

Significance testing was performed using the limma package (Linear Models for Microarray data) within R (Ritchie et al., 2015). Briefly, MaxQuant LFQ abundance values for each protein were log_2_ transformed, then quantile normalized, before a limma model was fitted. All possible treatment contrasts were performed and the eBayes function was used to calculate a moderated F-statistic of overall significance for each protein. *P*-values were also calculated and Benjamini–Hochberg correction for multiple-testing applied.

## Results and discussion

The MEROPS peptidase catalog for the *Fusarium* genome was utilized to provide the potential complement of peptidase genes. Four hundred peptidase-encoding genes were recorded in the *F. graminearum* genome by MEROPS (Rawlings et al., 2013). The majority of peptidases had serine (43%), metallo (28%), or cysteine (17%) nucleophiles. Threonine (6%), aspartic acid (5%), and glutamic acid (<1%) nucleophiles comprised the remainder of the peptidases (Table 2).

**Table 2:** **Peptidases identified from cellular and secreted proteomics data**.

**Peptidase nucleophile**	**Genome content**	**Cellular proteome**	**Secreted proteome**
Metallo	111	53	21
Cysteine	70	31	5
Serine	174	31	26
Threonine	22	20	12
Aspartate	20	6	7
Total	397	141	71

### An aggregated transcriptome profile for peptidases

**Figure 1:**
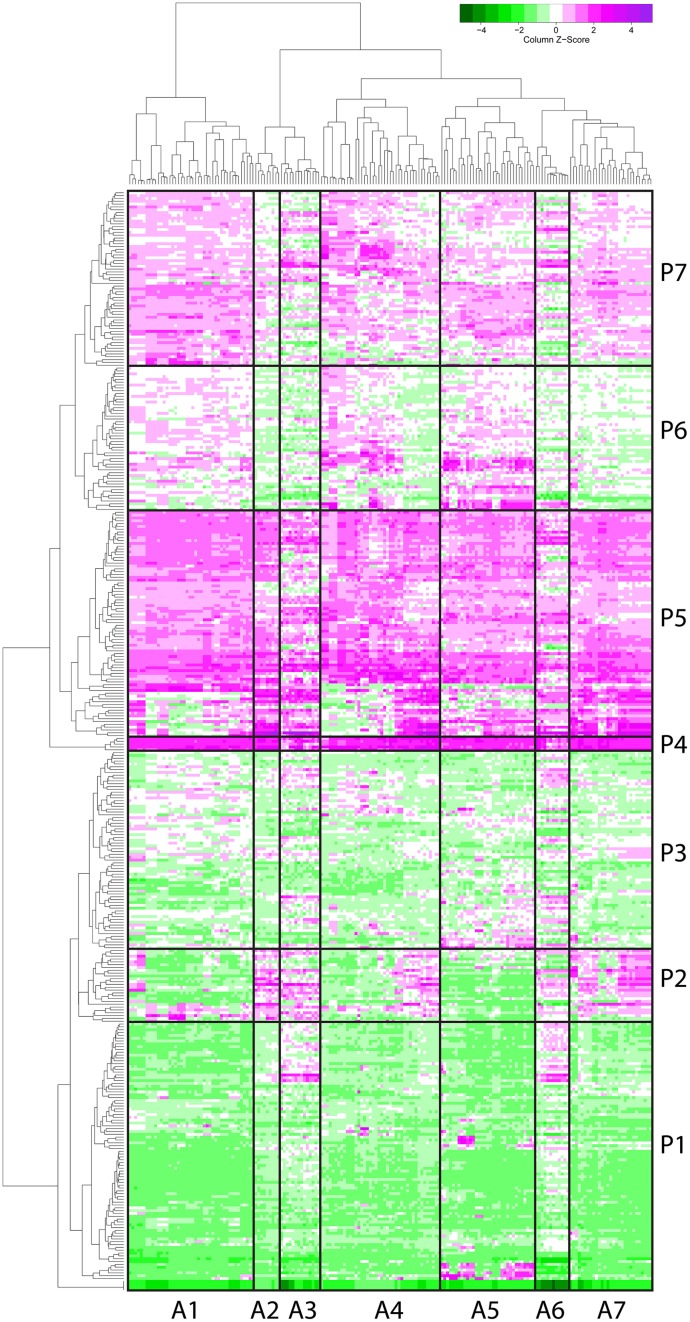
**Microarray heat map separates 389 peptidases into seven clusters based on their gene expression**. Gene expression profiles of proteases (by microarray) were clustered and a heat map built (magenta for above average expression, green for below average expression). A protease dendrogram is shown on the y axis, and a microarray dendrogram on the x axis. Clusters were generated from the dendrogram with manual refinement to produce seven clusters of arrays (A1–A7) and seven clusters of peptidases (P1–P7).

The *F. graminearum* affymetrix microarray platform and associated expression data were mined for a range of conditions capturing *in planta* disease, *in vitro* growth, sporulation, or mycotoxin production. Fourteen different experiments including 183 microarrays were combined to produce a transcriptomic profile for a total of 389 peptidases present on the array. A heatmap and dendrogram were calculated to group peptidases on the basis of their transcriptional expression profile (Figure 1, Table S1).

**Figure 2:**
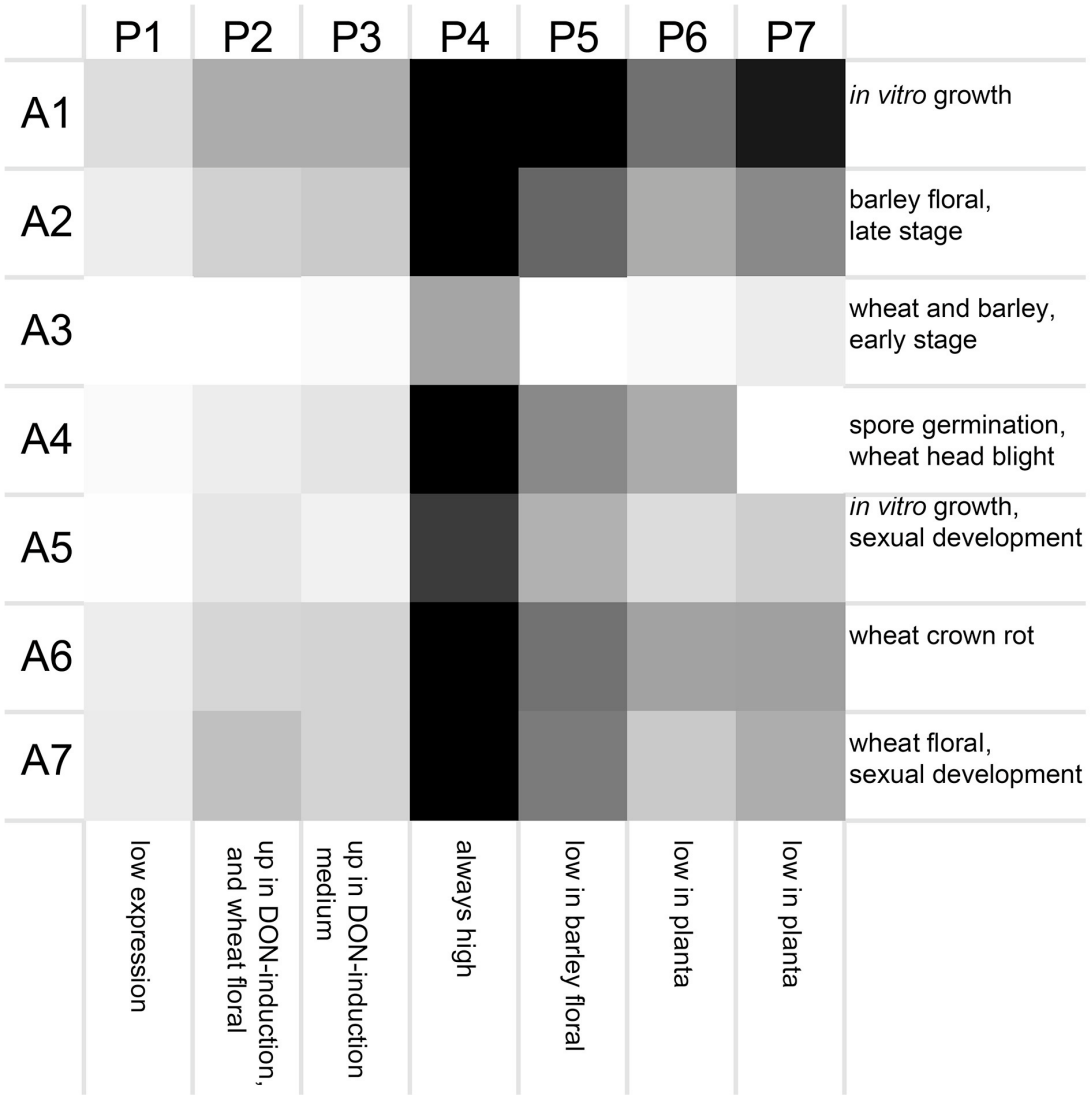
**Averaged gene expression profiles for seven clusters of peptidases**. Average values for peptidase (P1–P7) and microarray (A1–A7) clusters are plotted as a heat map (black, high expression; white, low expression). Characteristic treatments used in each array cluster are listed for each row, and characteristics of each peptidase expression profile are listed for each column.

Seven clusters of microarrays and seven clusters of peptidases were formed and average expression values calculated (Figure 2). The microarray experiments clustered largely as expected: array cluster A1 contained only *in vitro* mycelial growth arrays, A2 contained only barley floral infection arrays from 3 to 6 dpi, A3 contained mock inoculated controls and early stage floral infections for wheat and barley, A4 captured conidia germination *in vitro* and wheat head blight (2–8 dpi) and all wheat coleoptile infection arrays, A5 contained entirely *in vitro* growth arrays including sexual development on carrot agar and CMC medium, plus carbon starvation. A6 contained all of the wheat crown rot arrays, A7 contained only wheat floral infection arrays, including those tracking sexual development and all 4 dpi time points. These groupings were biologically relevant and distinguished growth on wheat vs. growth on barley, as well as sporulation and mycelial stages of growth, and growth during infections of crown and flowers.

The transcriptome profiles revealed two major groups of peptidases, high expression and low expression groups. Within those major types more refined clustering revealed peptidase genes that were regulated by environmental conditions, such as repression during *in planta* growth and those induced during mycotoxin biosynthesis. Peptidase cluster P1 had generally low expression, cluster P2 had higher expression during infection of flowers, and cluster P3 contained peptidases with medium-low expression. Cluster P4 and P5 had high expression. Expression in cluster P6 was generally low during growth *in planta*, and cluster P7 was high in both *in vitro* and in some *in planta* studies.

Peptidase nucleophile abundance varied between clusters (Table 3). Serine peptidases were overly represented in cluster P3 and to a lesser extent in clusters P1 and P2. Cysteine peptidases were enriched in cluster P6 and P7, while threonine and metallo peptidases were enriched in cluster P5. Aspartic peptidases were found in the smallest cluster, P4. Enrichment was not calculated for other rare nucleophiles due to insufficient numbers of peptidases in these categories. Serine peptidases were most commonly expressed at low to medium levels, while aspartic peptidases were generally transcribed constitutively at a high level.

**Table 3:** **Peptidase nucleophile type is enriched according to gene expression profile**.

**Peptidase cluster**	**Asp**	**Cys**	**Met**	**Ser**	**Thr**	**Total peptidases**
P1	0%	−6%	−1%	10%	–	95
P2	8%	−4%	−4%	6%	–	38
P3	–	−12%	−6%	22%	0%	55
P4	15%	3%	−8%	−4%	–	5
P5	−2%	−2%	8%	−14%	11%	80
P6	−1%	18%	−4%	−9%	−3%	51
P7	2%	9%	3%	−14%	1%	62
Total peptidases	19	66	108	172	21	386

*The peptidase nucleophile content of each gene expression cluster (Figure 1) was calculated and compared to the frequency in the genome. The percentage difference observed and expected frequency is shown for the five most common nucleophiles: Asp, aspartate; Cys, cysteine; Met, methionine; Ser, serine; Thr, threonine*.

The microarray analysis revealed that there was regulation of peptidase expression in response to growth *in planta*. We sought to further refine our analysis with proteomics analysis of the secreted proteome to determine the most abundant peptidases used for nutrient acquisition.

### A shotgun proteomics approach to identify secreted peptidases

A shotgun proteomics approach was used to identify extracellular peptidases of *F. graminearum* in culture. Using an ultra-high resolution linear ion-trap Orbitrap mass spectrometer an initial control sample of cellular proteins from a PDB culture produced over 2000 protein identifications via Peptide shaker software. This number reduced to 1743 robust identifications, with the requirement that each protein was matched with at least two validated peptides (Table 4, Table S2). The secretome was then queried through replicated analysis of four *in vitro* culture conditions: one complex plant-derived medium consisting of half-strength PDB and three defined media based on Czapek dox medium. A complete medium using peptone or tryptone was not included because we wanted to avoid pre-hydrolysis of proteins in the test medium. The defined media were identical except for substitution of the nitrogen source to one of nitrate (NO_3_), glutamine, or a nitrogen-free (Minus N) composition. These three sole nitrogen sources were chosen to examine an inorganic N source (NO_3_), an amino nitrogen source (glutamine) that provided both carbon and nitrogen, and a nitrogen-free medium to induce a nitrogen starvation response. An early-growth stage was selected to minimize cellular auto-lysis and mimic initial infection processes.

**Table 4:** **Proteins discovered in cellular and secreted treatments**.

**Growth medium**	**Fraction**	**Total proteins identified**	**Proteins only in the secreted proteome**	**Proteins only in the secreted proteome and predicted to be extracellular**
PDB	Secreted	217	128	75
CZ-NO3	Secreted	574	193	101
CZ-Minus N	Secreted	362	131	69
CZ-Glutamine	Secreted	515	212	114
PDB	Cellular	1743	N/A	N/A

**Figure 3:**
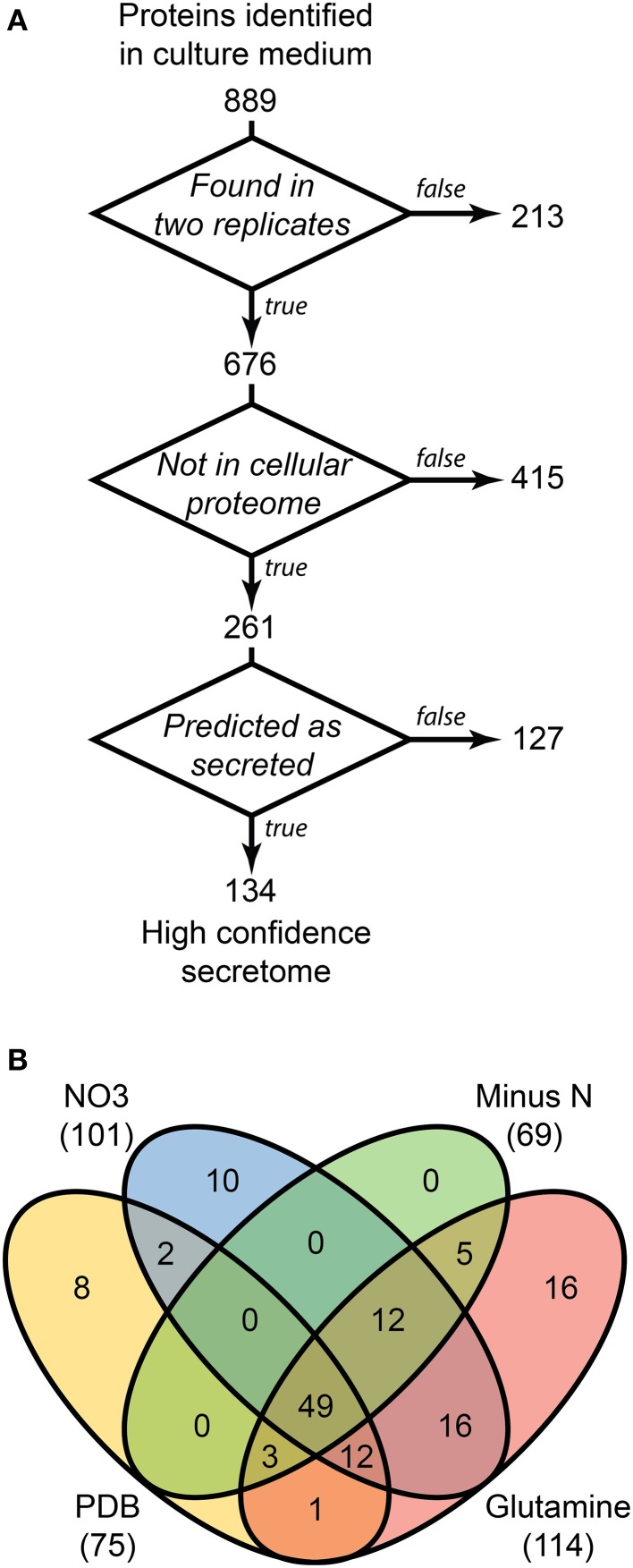
**Distribution of 134 proteins detected in secretome samples and absent from the cellular proteome**. An overview of the three-step selection process for the high-confidence secretome is shown in **(A)**. The distribution of protein identifications in the high-confidence secretome across the four *in vitro* conditions is shown in **(B)**.

LFQ of the secretome samples revealed large changes in protein abundance between treatments, which would have been unrecognized using more traditional qualitative assessments. MaxQuant LFQ identified 874 unique proteins across all secretome samples, with 676 present in a minimum of 2 of 3 replicates (Tables S3, S4). A further 261 of those were absent from the cellular control sample. We used a three-stage bioinformatics prediction to identify protein sequences with the correct signatures of secretion, a signal-peptide, lack of trans-membrane regions, and an extracellular location. Our bioinformatics prediction identified 668 proteins as likely to be secreted (Table S5). This number is higher than the 574 previously reported (Brown et al., 2012) due to our omission of a GPI-anchor prediction. The presence of a GPI anchor was considered insufficient evidence to exclude a protein from the secretome for two reasons. Firstly, the entirety of a GPI-anchored protein would still be external to the plasma membrane under our criteria, and secondly, GPI anchors may be cleaved to fully release proteins to the environment. When we added the bioinformatics filter to our 261 secretome proteins, just 134 remained (Figure 3A).

### A 134 protein high-confidence secretome

This strict filtering process should almost eliminate false positive secretome identifications. These 134 proteins formed the high-confidence secretome set. In total, 2025 unique proteins were found with high confidence in either the cellular sample or secretome samples. A previous study of a wide range of *in vitro* conditions and *in planta* apoplastic fluid identified 289 proteins (Paper et al., 2007). We have identified 153 of those 289 proteins including 43 previously considered *in planta*-only. By sampling the *in vitro* proteome to a deeper level, we have captured a significant proportion of proteins previously reported as *in planta*-specific.

The cellular proteome cohort had relatively fewer serine peptidases then the full genome prediction (22 vs. 44%), there were more metallo peptidases identified (38 vs. 28%) in the cellular proteome, and roughly the same percentage of cysteine peptidases in both groups (Table 2). The complement of exclusively secreted peptidases contained a high proportion of metallo peptidases. It is likely that some low abundance peptidases are missing from the proteomics datasets, which could influence the apparent cellular localization.

The PDB culture medium is complex and was likely to contain peptides of potato origin. An additional database search on a PDB culture filtrate was performed including the *Solanum tuberosum* predicted protein set v1.0.1 (The Potato Genome Sequencing Consortium, 2011), with the specific aim of assessing the residual potato peptide content in PDB medium. We identified three potato proteins in PDB grown samples, a starch synthase, a lipid transfer protein and a cytochrome b5 protein. We estimated that approximately 1% of the total detected peptides were of potato origin, and did not affect our analyses of *F. graminearum*.

Most of the secretome proteins were produced in more than one condition and 49 proteins were produced under all four conditions (Figure 3B). The defined medium with glutamine as the nitrogen source had the most proteins that were unique to one condition, whereas the Minus N condition had no exclusive proteins. Peptidases comprised 19 of the 134 protein high-confidence secretome, including five subtilizes, three aspartyl peptidases, three metallo-peptidases, and a trypsin peptidase.

### Nitrogen-responsive peptidases

**Figure 4:**
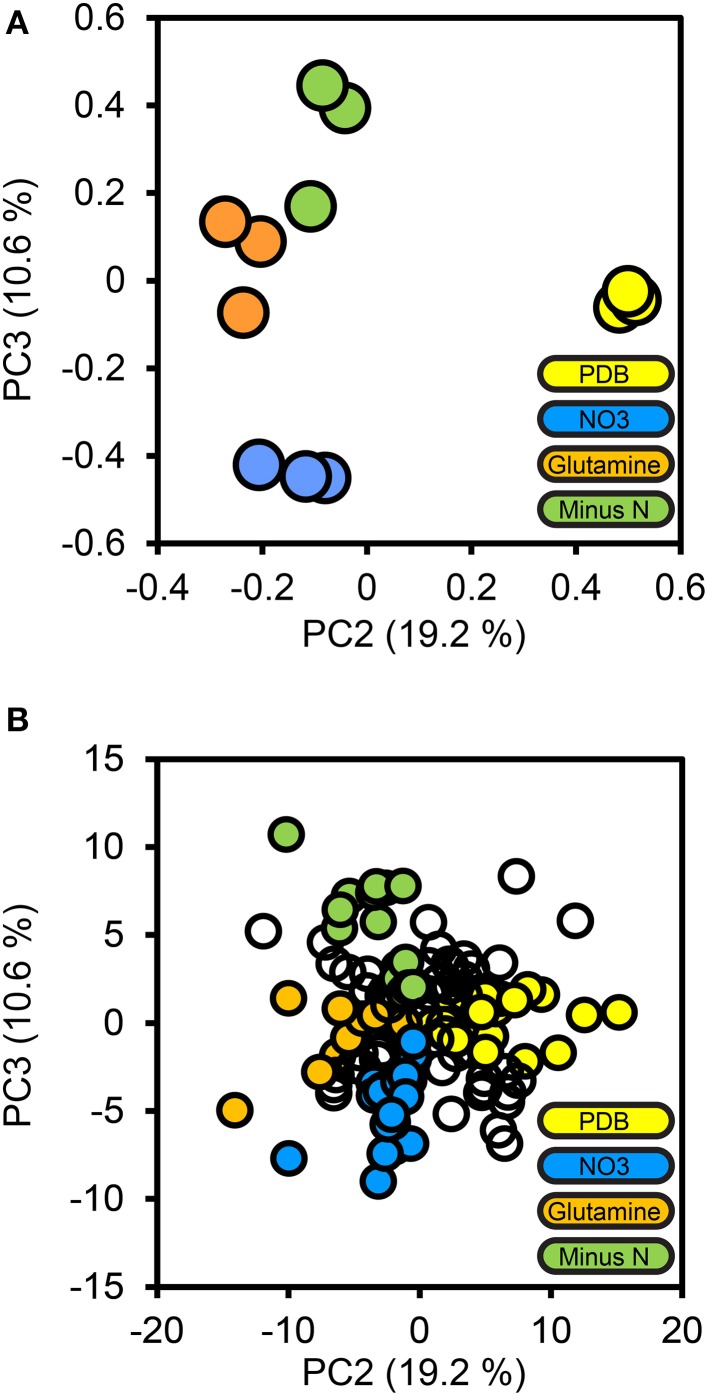
**Principal components analysis of the high confidence secretome**. Protein LFQ abundances for the 134 protein high confidence secretome subset were subjected to principal components analysis. The PCA scores plot for PC2 and PC3 are shown in **(A)**, with one marker drawn per biological replicate. The percentage of the total variance explained by each principal component is shown in brackets on each axis. Individual protein loadings for PC2 and PC3 are shown in **(B)**, with one marker per protein, proteins we considered to be most influential for each treatment medium are colored according to that medium, the remainder are drawn as empty markers.

To determine how protein secretion adapted in response to environmental nitrogen the high confidence secretome cohort of 134 proteins was subjected to PCA (Figure 4). Principal components 3 and 4 (PC3, PC4) captured 19.2 and 10.6%of the total variance, respectively, and resolved the four culture conditions tested. The loadings for PC3 and PC4 were examined to extract the most influential proteins for each culture condition, beginning with PDB. FGSG_08196 and FGSG_03072 were highly influential for the PDB treatment. FGSG_08196 is an example of the MEROPS scytalidoglutamic peptidase, an acid-active peptidase found throughout the ascomycete fungi. Inhibition of related glutamic acid peptidases of the thermophilic fungus *Talaromyces emersonii* significantly retarded hyphal growth on the complex nitrogen source peptone (O'Donoghue et al., 2008). We believe this is the first identification of FGSG_08196 in a proteomics study. A specific inhibitor of this peptidase may provide excellent control of *F. graminearum* and head blight disease.

FGSG_03072 a sedolisin, a part of the MEROPS S53 family of unassigned peptidases, a group containing both acid active endopeptidases and tripeptidyl-peptidases. FGSG_03072 aligned most closely (49% identity) with SedD, an acidic exopeptidase, and one of four characterized *Aspergillus fumigatus* sedolisins (Reichard et al., 2006). Blast searches for additional *F. graminearum* homologs of all the *A. fumigatus* Sed proteins revealed a SedA endopeptidase homolog (FGSG_10343) that was not detectable in the proteome, but a second SedB/C/D exopeptidase (FGSG_12142) was found in the high confidence secretome at high abundance in all four treatments. Interestingly, FGSG_12142 was not reported in previous proteomics studies on *F. graminearum* grown *in vitro, in planta*, during mycotoxin induction (Paper et al., 2007; Taylor et al., 2008; Rampitsch et al., 2010, 2012).

The minus-N culture medium contained four serine and one metallo peptidase in its influential set of proteins. FGSG_11164 (a Trypsin homolog), FGSG_10982 (dipeptidyl-peptidase), FGSG_03315 and FGSG_00806 (subtilisin-like peptidases), and FGSG_01818 (Ap1-like metallo-peptidase). All five of these peptidases were reported by Paper et al. (2007) from *in vitro* samples and only FGSG_10982 was not reported *in planta*.

The glutamine culture medium contained two influential peptidases. FGSG_03954, a metallo-endopeptidase, and FGSG_06572 a subtilisin-like serine peptidase. No peptidases were identified as being specific to the NO_3_ treatment; this is consistent with nitrogen catabolite repression during growth on a favorable inorganic nitrogen source. We expected equal repression of peptidases for all treatments containing favored nitrogen sources (NO_3_ and glutamine) this was the case for these two peptidases, as they were absent from the PDB treatment, where nitrogen catabolism should be de-repressed.

### Cross-referenced transcriptome and proteomics datasets

The peptidase transcriptome data and the proteome were cross-referenced. Cluster P2 of the transcriptome heat map was enriched with peptidases containing extracellular secretion signals. Twenty-nine per cent of P2 peptidases were predicted to be secreted, which was 14% more than the average for the genome. We did not observe enrichment of P2 peptidases in the actual secretome, which was probably due to their expression level falling below the limit of detection for our proteomics analysis. Peptidases in cluster P1 had the lowest average gene expression and this was again reflected in a very low number of identifications with only 7 of 97 P1 peptidases identified in the secretome or cellular proteome. This could be due to presence of pseudogenes that are not translated or more likely to low abundance proteins that fell below the limit of detection of MS/MS identification. The highly expressed clusters P4 and P5 were both enriched for peptidases that were identified in either the secretome or cellular proteome.

**Figure 5:**
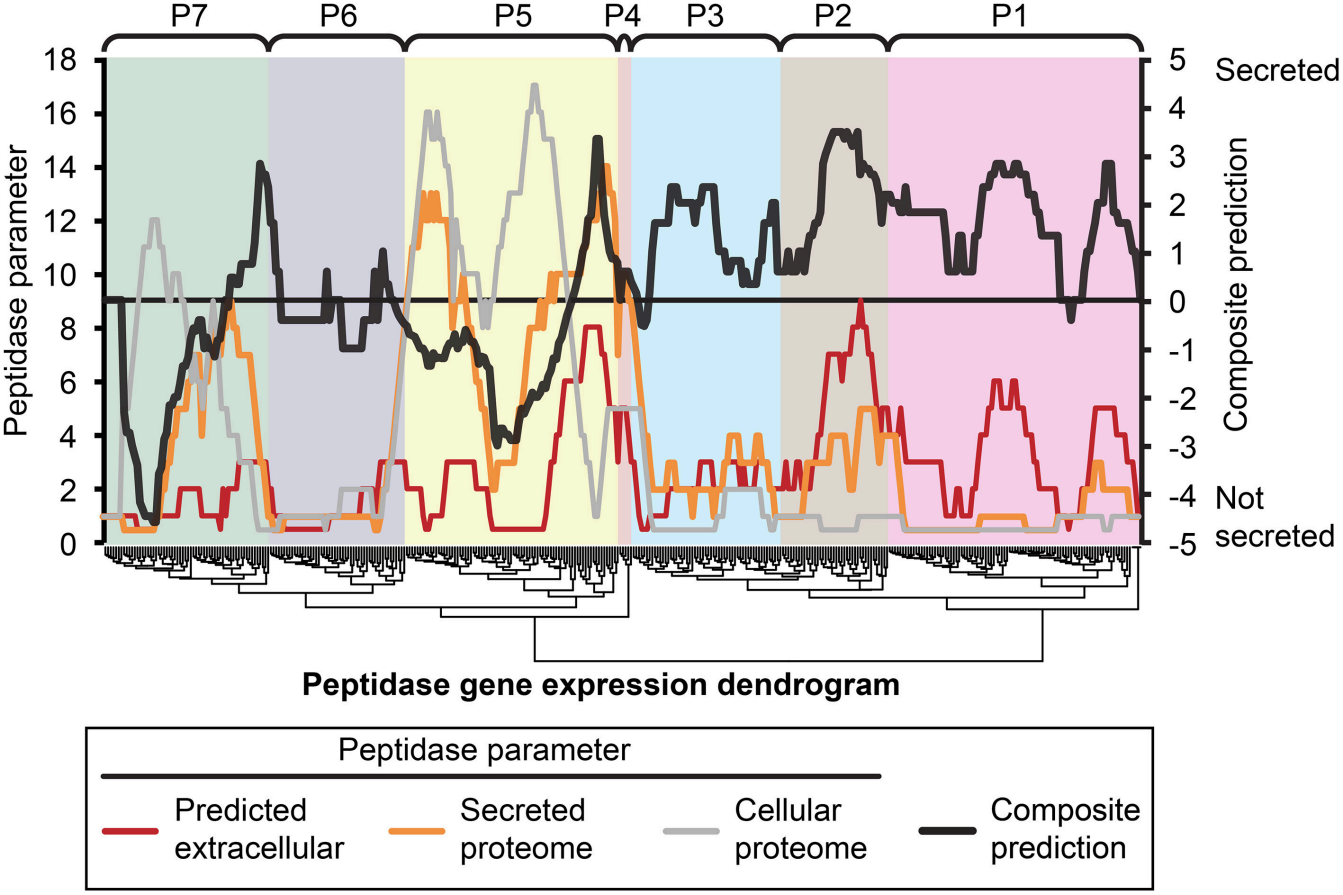
**Composite analysis of peptidase secretion and ***in planta*** expression**. Peptidase secretion parameters were calculated for each peptidase shown in Figure 1. Peptidases are ordered by their gene expression profile, as in Figure 1. The peptidase frequency for three peptidase parameters, (1) bioinformatic prediction of secretion “Predicted extracellular,” (2) presence in the “Secreted proteome,” and (3) presence in the “Cellular proteome” was plotted on the leftmost Y-axis axis as a moving window (window size = 16), for example, if the value is 10, that means 10 out of 16 peptidases in the window were predicted to be secreted based on sequence characters. The right-most Y-axis shows the resultant “Composite prediction” of secretion (black trace), Composite prediction was calculated as Log_2_[mean[(1),(2)]/(3)], where a positive result indicates more likely secretion, a negative result less likely secretion. The gene expression clusters of peptidases determined in Figure 1 are shown above the plot.

We hypothesized that peptidases upregulated during *in planta* growth would be enriched for signal-peptides and extracellular sequence signatures. Peptidases expressed during *in planta* growth were mostly found in clusters P2, P4, P5, while P6 contained peptidases that were mostly down regulated during growth *in planta*. We examined the peptidase gene expression heat map for clusters of peptidases that were likely to be secreted and also had similar gene expression profiles (Figure 5). Cluster P2 was almost uniformly predicted as secreted, while only a small subset of P5 was predicted to be secreted *in planta*. P2 peptidases were up-regulated during infection of barley and wheat flowers, crown rot of wheat, and sexual sporulation in wheat.

### Virulence factors for head blight of wheat and barley

The five peptidases influential for minus-N treatment were all classified within cluster P5 on the basis of their microarray profiles, which indicated generally high gene expression. The FGSG_08196 and FGSG_03072 peptidases identified in the PDB treatment were present in cluster P1, indicating low expression on average. Closer inspection of the arrays for each gene revealed a few conditions with elevated expression. FGSG_03072 was only highly expressed during growth on mycotoxin-inducing medium with agmatine or putrescine as a nitrogen source (FG7, FG14), and during sexual development *in planta* (FG16). FGSG_08196 was similar with selective high expression during nitrogen starvation, and during *in vitro* growth in mycotoxin-inducing media, although there was an additional peak in expression during barley floral infection at 72 h that was not repeated during infection of wheat flowers. Both peptidase types are active at acidic pH therefore we hypothesize their gene expression peaks in acidic environments. Mycotoxin-induction is known to occur during floral infections *in planta* and only at acidic pH *in vitro* (Merhej et al., 2011). The wheat floral tissue may develop a more acidic pH faster than barley, explaining why expression of these two acidic peptidases is higher during wheat infections. The pH of PDB culture medium is 5.1; this mildly acidic environment induced additional acidic peptidases compared to the NO_3_, minus N, and glutamine conditions. The three defined media were based on Czapek dox and have a neutral pH of 7.3. This may be sufficient to explain the increase in abundance of acid peptidases in the PDB treatment, and affirms the selection of PDB as an *in vitro* condition to approximate *in planta* growth.

### Comparison of head blight of wheat and barley

*F. graminearum* is a floral pathogen of both wheat and barley. Although this study did not collect *in planta* secretome data, we considered relationships between the *in vitro* secretome and the *in planta* transcriptomics datasets. Transcriptome data for both wheat and barley was examined for differential expression of proteases. Expression group A7 contained exclusively wheat infection samples, indicating there may be co-regulation of peptidases specifically *in planta*.

**Figure 6:**
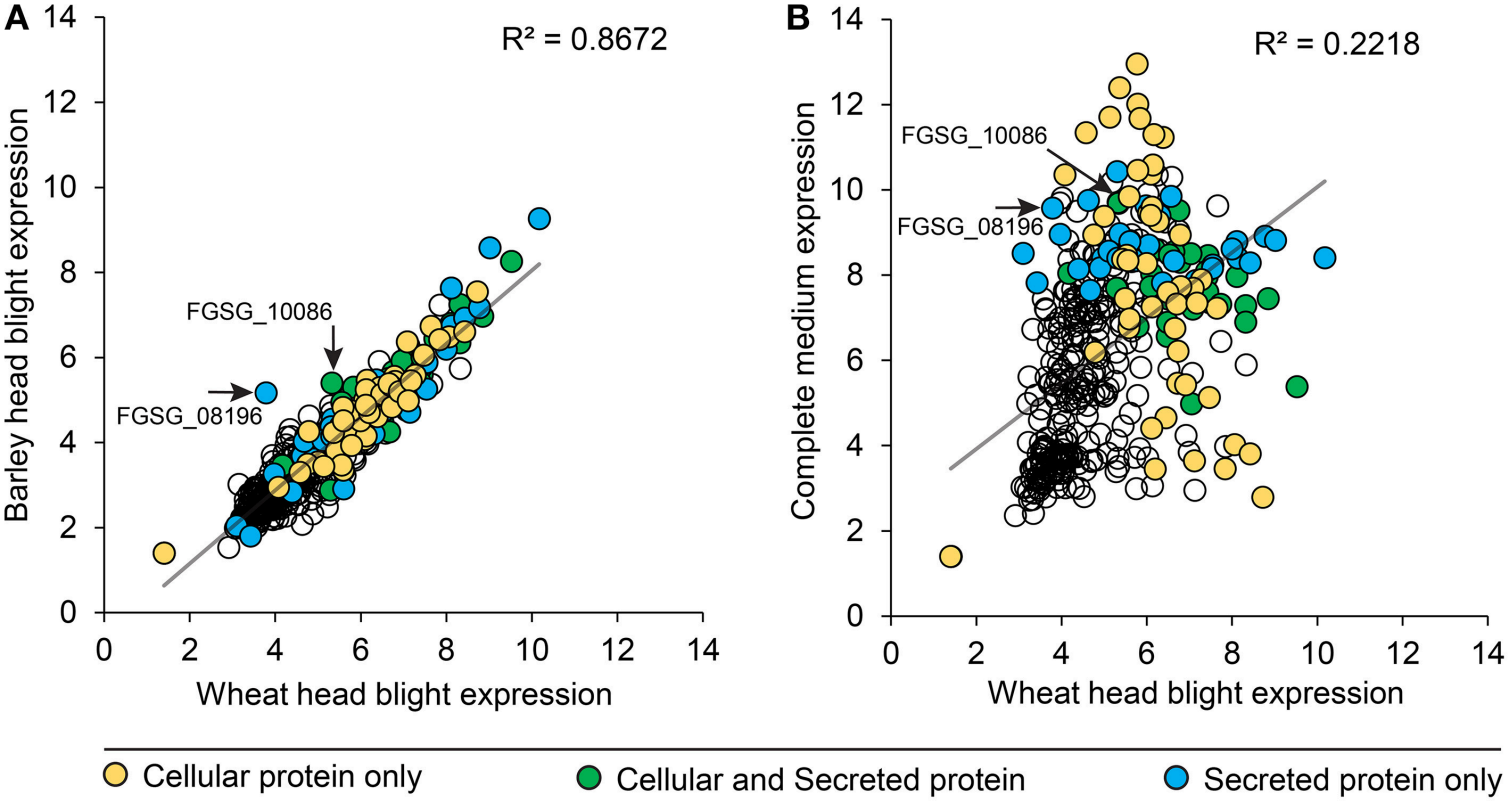
**Peptidase gene expression in wheat, barley and complete medium**. *F. graminearum* peptidase gene expression (RMA values) from microarrays of barley and wheat head blight disease **(A)** were plotted to reveal the degree of transcriptional gene co-regulation on the two different host plants. Two peptidases that were differentially reguated between the two *in planta* conditions are indicated with an arrow and label. For comparison, gene expression (RMA values) from *in vitro* growth on complete medium were compared to wheat head blight **(B)**. The secretion status determined from proteomics analyses is displayed in the shading of each peptidase value: yellow for cellular proteins, green for proteins found in both the cellular and secreted proteomes, and blue for proteins only found in the secreted proteome.

We compared peptidase gene expression in barley and wheat microarrays to determine if peptidase expression was regulated by the species of host plant (Figure 6). A range of expression levels was identified, but in the vast majority of cases average peptidase expression was consistent between the two host plants with a correlation coefficient (R^2^) of 0.8672 (Figure 6A). Such high correlation is impressive considering the independent nature of the experiments. To confirm that peptidase gene expression can be modulated to suit the environment, we compared wheat infection to *in vitro* growth on complete medium (Figure 6B). We observed large changes in peptidase expression when comparing *in vitro* with *in planta* growth, with a correspondingly weak correlation coefficient of 0.2218. This confirmed that peptidase transcription is responsive to the growth environment. Peptidases identified in either the cellular proteome, secreted proteome or both cellular and secreted proteomics samples were mapped onto the correlation charts. No overall patterns of secretion were revealed in relation of gene expression level on barley or wheat, however, secretome peptidases tended to be transcribed more during growth on complete medium than on wheat. The secreted peptidases FGSG_08196, and FGSG_10086 were revealed as slightly up-regulated during barley infection compared to wheat infection. FGSG_8196 was identified in the 134 protein high confidence secretome, and was exclusively detected in the PDB medium secretome. FGSG_10086 is a serine peptidase in the MEROP S33 family in gene expression group P3, most highly expressed in mycotoxin induction medium.

### Non-canonical secretion

Non-canonical secretion mechanisms may account for unexpected extracellular location of proteins. This study focussed on secreted peptidases, some of which may have been incorrectly regarded as cellular proteins due to non-canonical secretion. We identified 127 proteins that are candidates for non-canonical secretion as they were only found in the secreted proteome but lacked the expected bioinformatic prediction of secretion (Figure 3A). This figure is likely an overestimate as our bioinformatics methodology was biased toward false-negative error when assigning classical secretion, but 47 of those 127 proteins have a SignalP score of less than 0.15 (the threshold for secretion is 0.45), comprising a more representative list for non-canonical secretion. Superoxide dismutase is a good candidate for non-canonical secretion as it has been reported to be released into culture medium by gentle washing of *Claviceps purpurea* hyphae, yet lacks a classical signal peptide (Moore et al., 2002). Non-canonical secretion of proteins via extracellular microvesicles, or exosomes, is gaining attention for its potential role in cell-to-cell communication and pathogenesis (Samuel et al., 2015). We identified three homologs of superoxide dismutase in the culture medium proteome, FGSG_02051, FGSG_04454, FGSG_08721. All three were absent from PDB medium and were present in the other three treatments. None contained recognizable signal peptide sequences and two were also found in the cellular proteome, FGSG_08721 has been previously reported as secreted both *in vitro* and *in planta* (Paper et al., 2007). Nine of the 127 candidates for non-canonical secretion were peptidases, including four metallo-peptidases of the M28 family. Of these, FGSG_01095 and FGSG_11411 had extremely low signalP scores of 0.131 and 0.103, respectively. Interestingly, the FGSG_01095 sequence scored higher using the prokaryotic SignalP algorithms, raising the question of whether proteins originating from mitochondria have retained aspects of prokaryotic protein transport.

### Mycotoxin biosynthesis and protein secretion

Brown et al. (2012) suggested that there may be a link between symptomless growth *in planta* and the co-secretion of trichotheces (including deoxynivalenol, or DON) and virulence proteins. Both their study and this one identified the deoxynivalenol biosynthetic enzyme TRI8 (trichothecene 3-O esterase, FGSG_03532) as a predicted secreted protein on the basis of its sequence characters. However, we did not identify it in our proteomics survey. This was unsurprising, as deoxynivalenol biosynthesis by *F. graminearum* requires a low pH, a permissive nitrogen source such as a polyamine or N-starvation. The transcriptional regulator AREA governs genes required for nitrogen metabolism and is also required for full DON biosynthesis in *F. graminearum* (Hou et al., 2015). The key biosynthetic and regulatory genes, *TRI5* and *TRI6*, respectively, are induced under nitrogen starvation conditions, and supressed by a preferred nitrogen source, such as ammonia. The culture period of our study was very short and would not have resulted in significant DON induction under permissive conditions. Taylor et al. (2008) specifically targeted mycotoxin-induction for their ITRAC proteomics analysis of cellular proteins, and identified three TRI proteins, FGSG_03534, FGSG_03535, and FGSG_03543. We did not find any of the TRI proteins in our cellular controls, nor our secreted protein samples, which may have been due to our early-stage sampling of cultures. We expect that there may have been low levels of TRI proteins in our MinusN sample but they were insufficient for MS/MS detection from culture medium. Deoxynivalenol permissive conditions would also result in vigorous expression of secreted peptidases, which we observed in the MinusN proteomics samples. It may be possible to use the presence of certain secreted peptidases as a highly sensitive enzymatic reporter of deoxynivaleol risk in cereal grains.

## Conclusion

This is the first proteomics study to focus on the peptidases of *F. graminearum*. Degrading enzymes are considered diverse and redundant, and therefore unlikely targets for control of plant pathogens. However, our characterization of the secreted peptidases of *F. graminearum* revealed deployment of a greatly reduced peptidase subset of limited diversity. We identified a *Fusarium* homolog of a peptidase required for hyphal growth of a thermophilic fungus, this homolog presents a target for further study to determine its contribution to overall fitness of *F. graminearum*, and a possible control target.

We have brought together public transcriptomics resources and an *in vitro* secreted proteomics dataset to extend our knowledge of peptidase production of *F. graminearum* both *in planta* and *in vitro*. A focussed peptidase gene expression analysis revealed seven clusters of peptidases with similar expression profiles during *in vitro* and *in planta* conditions. Over 2000 proteins were identified with 890 of those released into culture medium. A high-confidence secretome cohort of 134 proteins was derived that satisfied a three-stage selection process: firstly, presence in the culture medium, secondly, absence from the cellular proteome, and thirdly, satisfaction of three bioinformatics analyses for secretion characters. High sensitivity mass-spectrometry analysis of *in vitro* extracts allowed extension of the known proteome of *F. graminearum*. The majority of the high confidence secretome had not been reported in previous proteomics studies and includes proteins previously thought to be restricted to *in planta* growth. We anticipate this dataset will also allow future refinement of the *F. graminearum* genome annotation, confirming post-transcriptional processing, and N-termini of mature proteins.

## Author contributions

RL, MB, MA drafted the manuscript. OM, CC, PF, RL performed experiments. MB, RL, MA, SM conceived the experiments. All authors edited and reviewed the manuscript.

## Funding

This work was supported by the Australian Research Council with Discovery Projects to MA (DP150104386), and SM (DP130100535), plus a Discovery Early Career Researcher Award (DE150101777) to SM, and a La Trobe University Understanding Disease Research Focus Area Grant to MB.

### Conflict of interest statement

The authors declare that the research was conducted in the absence of any commercial or financial relationships that could be construed as a potential conflict of interest.

## Footnotes

^1^Fusarium Comparative Sequencing Project, Broad Institute of Harvard and MIT [WWW Document], n.d. URL http://www.broadinstitute.org/.

## Supplementary material

Table S1 **Peptidase microarray data**.

Table S2 **Cellular proteome identifications**.

Table S3 **Label-free quantitation**.

Table S4 **Limma statistical analysis**.

Table S5 **Signal peptide predictions**.
